# When I feel that I am better off, science seems to make the world better off too: inequality, perceived standard of living and perceptions toward science

**DOI:** 10.3389/fpsyg.2023.1202550

**Published:** 2023-10-05

**Authors:** Seungwoo Han, Yookyung Lee

**Affiliations:** ^1^Center for Digital Humanities and Computational Social Sciences, Korea Advanced Institute of Science and Technology (KAIST), Daejeon, Republic of Korea; ^2^College of Engineering, Chung-Ang University, Seoul, Republic of Korea

**Keywords:** inequality, perception of living standards, polarization over science and technology, COVID-19, Global North and South

## Abstract

The COVID-19 pandemic has underscored that divisive views on science and technology span both the Global North and South. This study posits that an individual’s perception of their current living standard acts as a mediating factor linking income inequality to attitudes towards scientific and technological advancements. It contends that rising income disparities shape perceptions, making individuals feel their current living conditions have not surpassed those of prior generations. Consequently, such perceptions diminish the likelihood of recognizing the positive impacts of science and technology on societal progress and future prospects. This paper sheds light on how escalating inequality fosters societal rifts concerning science and technology.

## Introduction

The landscape of public opinions on science, health, and technology has long been a focal point of significant academic interest. Much of the existing literature has focused on the Global North, emphasizing particularly salient and, at times, contentious topics such as vaccinations, viruses, climate change, and the swift advancements in artificial intelligence (Krause et al., 2019; Sulik et al., 2021; Schmid et al., 2022; Meyer, 2023; Romer and Jamieson, 2023). These high-stakes issues have elicited a spectrum of responses from the public, ranging from agreement to polarization. However, the onset of the COVID-19 pandemic has broadened the academic perspective to also spotlight pronounced divisive opinions in the Global South (Matos et al., 2022). This shift highlights a crucial insight: skepticism or trust in scientific undertakings is not merely a phenomenon confined by geographical boundaries or a nation’s economic standing. Instead, this study suggests that such attitudes can be seen as a universal human reaction, deeply connected to socio-economic patterns, specifically economic inequality, prevalent across diverse societies.

Interestingly, while the latter half of the 20th century witnessed a substantial alleviation of global poverty and a narrowing of economic disparities between nations, the internal fabric of individual countries narrates a different story. Inequalities within countries, both developed and developing, have surged (Chancel and Piketty, 2021). This presents a paradox: global progress has not necessarily translated into universally perceived individual prosperity. For many, their personal living standards might feel stagnant or even worse off when juxtaposed against the backdrop of their parents’ or grandparents’ generations.

This observation prompts a crucial line of inquiry: is an individual’s faith in the potential of science and technology intricately linked to their subjective evaluation of their life quality? If one does not perceive a personal upliftment over generations, despite overarching societal progress, would they inherently doubt the promises of scientific and technological advancements?

The academic community has extensively explored the polarization of perceptions surrounding science and technology. Research has delved into the impacts of media representation (Huber et al., 2019; Hameleers and Boukes, 2021; Gurevich, 2022; Fleury-Bahi et al., 2023; Hong, 2023), political ideologies, religious beliefs (McCright et al., 2013; Agarwal et al., 2021; Akin et al., 2021; Jiang et al., 2022), and broader socio-economic determinants (Achterberg et al., 2017; Agarwal et al., 2021; Han and Lee, 2022; Baker and Merkely, 2023). However, there remains a gap in understanding the effects of inequality.

This study endeavors to fill that void, venturing into the relatively uncharted territory of how personal experiences within unique socio-economic contexts mold one’s outlook on science and technology. We propose a hypothesis that is both simple in its conception and profound in its implications: perceptions of living standards, especially when set against the living conditions of previous generations, act as a potent mediator. They bridge the vast expanse between structural income inequalities and collective attitudes towards the forward march of science and technology.

To truly capture the nuances of this relationship, our analysis takes a dual-pronged approach. We first aim to delineate the intricate links between prevailing income inequality patterns and personal assessments of living standards. This foundational understanding then sets the stage for the second, and arguably more critical, phase: determining how such perceptions shape attitudes towards science and technology.

In synthesizing these threads, we aspire to paint a comprehensive picture, one that nuances the multifaceted dynamics between economic structures, personal perceptions, and the broader societal views on scientific and technological progression. Our findings not only enrich the current academic dialogue but also present a fresh lens through which we can understand the increasingly polarized world views in this domain.

## Inequality, perception of living standards and science and technology

### Understanding socio-economic perceptions: a journey from past to present through the lens of inequality

Humans inherently employ social comparison as a tool to decode relational dynamics within their environment. Rooted in Tajfel’s (1981) work, this strategy extends beyond mere interpersonal assessments, deeply entwining with one’s subjective understanding of their socio-economic position. Within these evaluations, individuals continually seek benchmarks, often aligning their socio-economic standing in relation to peers (Evans and Kelly, 2004). Contemporary research, such as that by Fernández-Albertos and Kuo (2018) and Newman et al. (2015), suggests that such comparative elements can wield considerable influence over public opinion. Amplifying this claim, studies by Fraile and Pardos-Prado (2014) and Gimpelson and Treisman (2017) highlight that broader societal perspectives often stem from individual appraisals of economic circumstances, anchored in these comparative frameworks. Delving further, spatial contexts, highlighted by Han and Kwon (2023a) and Szewczyk and Crowder-Meyer (2022), provide another influential layer, steering these comparative judgments and consequently shaping attitudes.

However, the scope of these comparisons is not confined to the present. An inherent human inclination is to gauge the current against the backdrop of the past, frequently drawing upon prior generations as reference points. Such intergenerational evaluations form a critical lens through which societal advancements are perceived. A classic exposition of this phenomenon is the ‘Easterlin Paradox’, where Easterlin (1974) posits that beyond a certain point, economic progress does not directly engender heightened subjective well-being. This idea resonates with the prevalent tendency to contextualize one’s existing socio-economic realities against ancestral templates. Adding depth to this dialogue, Kahneman and Deaton (2010) articulate that sheer income augmentation does not necessarily culminate in heightened emotional contentment. The real influencer, they contend, lies in perceptions stemming from juxtaposing the now with the past. Clark et al. (2008) build on this, exploring the interplay between relative income and contentment, spotlighting the profound implications of these socio-economic alignments, particularly when weighed against bygone eras. A complementary perspective is offered by Giuliano and Spilimbergo (2014), positing that impactful macro-economic occurrences, like recessions, leave indelible marks on the psyche of the impacted generation, especially when contrasted with antecedent generations’ experiences. Such contrasts invariably shape contemporary perceptions, preferences, and choices.

Income disparities, as elucidated by Kuhn (2020), are intricately woven into the fabric of daily experiences. Rather than being mere abstract concepts, these inequalities, when contextualized within day-to-day economic frameworks, magnify an individual’s consciousness of their socio-economic position. This is because, fundamentally, inequality is relational in nature (Cavacho and Álvarez, 2019; Condon and Wichowsky, 2020). A prominent manifestation of escalating income disparities is “status anxiety,” a psychological condition prevalent across all income tiers but especially intensified in starkly unequal societies (Layte and Whelan, 2014; Rodríguez-Bailón et al., 2020). This anxiety encapsulates fears tied to one’s social ranking, ranging from not meeting societal benchmarks of success to concerns of socio-economic stagnation or even decline (De Botton, 2004). Such inequalities lead individuals to keenly evaluate their socio-economic status in comparison with past and present generations. The mechanics behind this observation will be further discussed in the following subsection.

### Socio-economic perceptions influence on attitudes towards scientific and technological advancements

Bandura (1978) emphasizes self-efficacy’s significance as a driving force behind learning and acquiring new skills. A wealth of literature further supports its role in fostering individuals’ recognition of the efficacy of science and new technologies, leading to their integration into daily life (Bandura, 1978; Gist and Mitchell, 1992; Compeau and Higgins, 1995; Scherer et al., 2019). High self-efficacy is associated with setting challenging goals, persevering in the face of obstacles, and investing greater effort in mastering complex subjects (Zimmerman, 2000; Pajares and Schunk, 2001; Schunk and Pajares, 2002). Additionally, individuals with strong self-efficacy perceive technology as more manageable and are more inclined to adopt and integrate it into their lives, thereby facilitating widespread acceptance of new technologies (Compeau and Higgins, 1995; Agarwal and Prasad, 1999; Venkatesh and Davis, 2000; Scherer et al., 2019). However, it is important to acknowledge the role of external factors, such as social support and feedback, in influencing the recognition of the effectiveness of science and new technologies (Betz and Hackett, 1983; Bandura, 1986; Lent and Hackett, 1987). In essence, self-efficacy, along with its interaction with external factors, plays a pivotal role in understanding the advancement of science and technology. Despite recognizing the impact of external elements, self-efficacy remains relevant and holds its significance within this context.

Given this backdrop, if individuals gauge their life quality as stagnant or regressed vis-à-vis their forebears due to economic disparities, even in the face of evident advancements, it could cripple their self-efficacy pertaining to absorbing novel knowledge, especially in scientific and technological domains. Essentially, this suggests a potential dilution in discerning the present utility and futuristic potential of these domains. If, subjectively, they remain oblivious to socio-economic strides, they might not perceive scientific and technological leaps as societal enhancers or as harbingers of prospective boons.

Furthermore, the saliency and immediacy of everyday occurrences color perceptions profoundly (Tversky and Kahneman, 1974). Although society might objectively flourish, courtesy of scientific and technological breakthroughs, personal experiences, especially when adverse, hold the potential to cloud judgment. The inadvertent oversight of privileges or the luxury of “possession,” can birth scenarios where the singular adversity overshadows a sea of conveniences (McIntosh, 2007). Consequently, if lived experiences do not resonate with perceived betterment in life quality, regardless of objective headways, there’s an inherent risk of diminishing self-efficacy in science and technology, potentially prompting a dismissal of their contributions.

The examination of how perceptions are shaped by a combination of factors, including social relationships and the socio-economic environment related to inequality, emerges as a crucial aspect in understanding public opinion regarding the role of science and technology. In particular, the exaggeration of individual experiences can potentially amplify this tendency. These discussions underscore the significance of considering individuals’ perceptions of the role of science and technology in light of their unique socio-economic context and personal experiential background. By recognizing the multifaceted nature of these influences, this section offers a comprehensive understanding of the intricate relationship between public opinion and science and technology.

## Empirical assessments

This section sequentially presents two empirical investigations, as part of a thorough assessment of the hypothesis, by forming a macro-to-micro connection. The first investigation analyzes how income inequality affects perceptions of living standards, in comparison to previous generations. The second investigation examines how these perceptions influence attitudes towards the impact of scientific and technological advancements, with respect to the present and future. The first analysis is conducted at the macro level, while the second analysis is conducted at the individual (micro) level. Both analyses share a key variable, that is, perception of one’s standard of living, as compared to one’s parents’. These two sequential analyses supplied evidence for this research’s argument.

### Analysis 1

This study aims to examine the relationship between income inequality and perceptions of living standards compared to those of one’s parents’ generation. It utilizes macro-level data from the World Values Survey (WVS) Wave 7 (2017–2020) for 46 countries, with a total of about 42,000 observations. See Appendix A for a list of countries included in the analysis and descriptive statistics. The WVS is an academically driven, cross-country collaboration for nationally representative surveys. The national average of perceived standard of living is used as the dependent variable, with the question “If you have to compare your standard of living with that of your parents, when they were about your age, would you see yourself as better or worse off?” and answers ranging from “Worse off (=1)” to “Better off (=3).” The primary explanatory variables for the analysis are the Gini index, Top 1% income share, Top 10% income share, and Ratio of Top 10% income share to Bottom 50% income share. These variables are obtained from the World Inequality Database and measured based on pre-tax and pre-transfer income. GDP *per capita* is also included as a control variable, with a log transformation applied to it.

**Figure 1:**
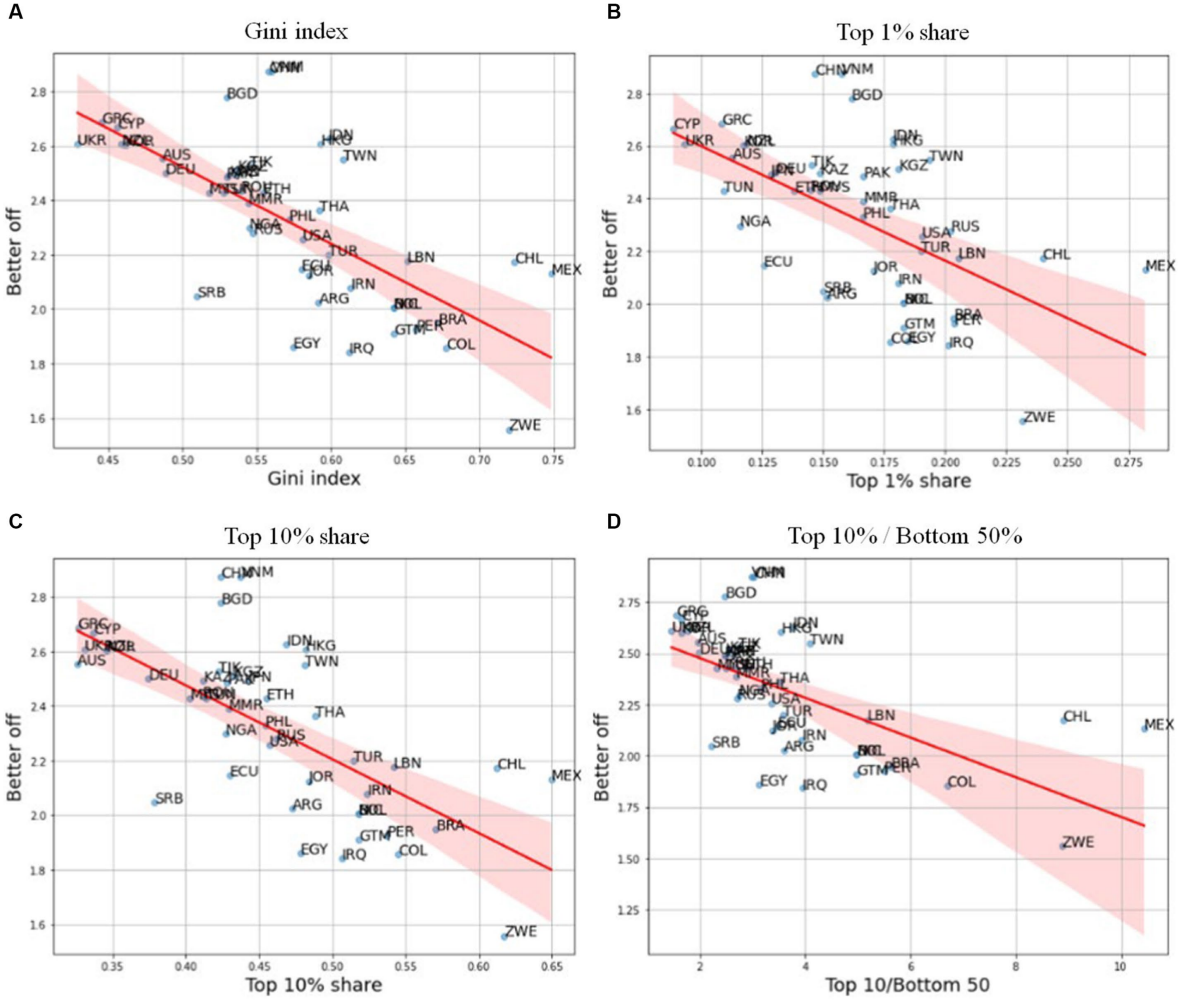
Income inequality, and perceptions of living standards as compared to one’s parents. Source: better off from WVS Wave 7; income inequality variables are from world inequality database. **(A)** Gini index. **(B)** Top 1% share. **(C)** Top 10% share. **(D)** Top 10%/bottom 50%.

Before seeing the empirical assessments, we can begin with descriptive analysis in Figure 1. Figure 1 presents the intuitive relationship between income inequality, and perceptions of living standards. This simple and intuitive measurement framework is suitable for an empirical approximation of individual perceptions of income inequality (Kuhn, 2020). It is clear that the perceived standard of living is highly and negatively correlated with various estimates of income inequality, with regard to the Gini index, income share, and ratios of the top and bottom income shares. This descriptive analysis allows inferring that the formation of public perceptions of living standards, by comparing with previous generations, incorporates at least some information on real economic outcomes. In Supplementary Figure B1 in Appendix B, countries are divided into groups of high and low income inequality on the basis of median value. Based on this, Supplementary Figure B1 presents the kernel density estimation (KDE) for each group. The KDE results show that, perceived standard of living is distributed at lower values in countries with high income inequality, and at higher values in countries with low income inequality.

Table 1 includes four regression models, which compare the effects of income inequality measures, and perceptions. All income inequality variables are found to be statistically significant in a negative direction. As income inequality increases, people are more likely to think that their standard of living is worse than that of their parents. To explain this statistically, an increase in the Gini index, and alternative measures of income inequality, such as income ratios of the Top 1%, Top 10% and Top 10%/Bottom 50%, raises individuals’ concerns about standard of living. It is noteworthy that various measures of income inequality provide the qualitatively same, consistent results for this association. The effect of GDP *per capita* is not statistically significant in all models. In other words, perceptions do not seem to be closely related to macro-economic conditions, such as GDP *per capita*.

**Table 1:** Income inequality, and perception of living standards, as compared to one’s parents’.

	DV: Better off comparing with parents’ generation			
	(1)	(2)	(3)	(4)
Gini index	−2.727*** (0.434)			
Top 1% share		−4.154*** (0.803)		
Top 10% share			−2.629*** (0.449)	
Top 10/bottom 50				−0.092** (0.024)
ln GDP p.c.	0.03 (0.039)	0.062 (0.043)	0.026 (0.04)	0.045 (0.037)
Intercept	3.588*** (0.533)	2.411*** (0.468)	3.273*** (0.516)	2.223*** (0.414)
*R* ^2^	0.47	0.25	0.22	0.24
*N*	46	46	46	46

Country clustered standard errors in parentheses: *p < 0.05, **p < 0.01, ***p < 0.001.

### Analysis 2

We conduct an individual-level analysis using the WVS Wave 7 data, which comprises approximately 42,000 observations from 46 countries, to examine how perceptions of living standards influence beliefs about scientific and technological advancements. The primary explanatory variable is perceptions of living standards as compared to one’s parents’, which ranged from 1 to 3 and is the same variable used in the previous analysis. The dependent variables are (1) whether individuals believed the world is better or worse off due to science and technology, and (2) whether they believe there are more opportunities for the next generation because of science and technology. The first dependent variable is measured using a question with ten answer categories ranging from “A lot worse off (=1)” to “A lot better off (=10).” The second dependent variable is measured using a question with ten answer categories ranging from “Completely disagree (=1)” to “Completely agree (=10).” To control for socio-economic and demographic characteristics, a set of variables is included in the analysis. Further details can be found in Supplementary Table A2 in Appendix A. Furthermore, to control for possible country-specific variations, we employ fixed country effects estimations. Notably, perceptions of living standards can differ across countries; therefore, including country fixed effects allows us to address these variances.

Before considering the estimations results, let us begin with a descriptive analysis in Supplementary Figure B2 in Appendix B. Supplementary Figure B2 suggests that individuals hold different views about scientific and technological advancements, and their contribution to current progress in society, as well as future opportunities. On an average, people who believe that their standard of living is better than that of their parents perceive science and technology as having improved the world, and will provide more opportunities. This suggests the need to investigate the association between one’s perception of living standards, and of contributions made by science and technology.

We now move to the main analysis in Table 2. The main analysis is presented in Table 2, where four models are used. In Models (1) and (3), the reference group is “About the same,” and as a result, the variables of “Worse” (=1) and “Better” (=3) are included. On the other hand, in Models (2) and (4), the variable of “Better off,” ranging from 1 to 3, is included. The results of the analysis show that the regression coefficients of “Worse” are negative and statistically significant in Models (1) and (3), while the regression coefficients of “Better” and “Better off” are positive and statistically significant, providing strong support for the argument. In other words, the more individuals perceive their living standards to be worse than those of their parents’ generation, the more they perceive science and technology as having a negative impact on their present living standards and future opportunities. This finding suggests that if individuals do not believe that their lives are better off than their parents’ generation, they are more likely to view science and technology as not having improved their living standards and failing to bring about future opportunities.

**Table 2:** Perceptions of standard of living, and science/technology.

	DV: Better off because of science and technology		DV: More opportunities because of science and technology	
	(1)	(2)	(3)	(4)
Worse	−0.259*** (0.051)		−0.246** (0.065)	
Better	0.208*** (0.049)		0.19*** (0.031)	
Better off		0.229*** (0.026)		0.213*** (0.027)
Intercept	5.963*** (0.129)	5.488*** (0.131)	6.53*** (0.16)	6.086*** (0.135)
Controls	√	√	√	√
Country FE	√	√	√	√
N	42,845	42,845	42,808	42,808

Ordinary least squares estimations with country fixed effects and country clustered standard errors are applied as the analytical model for intuitive interpretation. The results of the multilevel ordered logit model can be found in Appendix C and are largely qualitatively similar to the results presented in this section. Controls are not reported. See Supplementary Table B1 for full results. Country clustered standard errors in parentheses: * *p* < 0.05, ** *p* < 0.01, *** *p* < 0.001.

**Figure 2:**
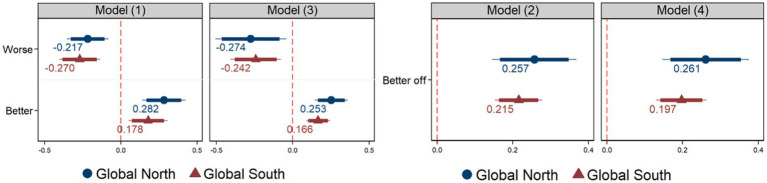
Perception of living standards, and science and technology, in the Global North and South. Controls are not reported. See Supplementary Tables B2, B3 for full results. Coefficient plots with 90% (thick line) and 95% (thin line).

The following analysis examines the differences in perceptions of science and technology between the Global North and South, where the Global North is defined as high-income countries such as the United States and Germany by the World Bank. The analysis, presented in Figure 2, shows that attitudes towards science and technology are polarized in both regions. Interestingly, the analysis finds no significant difference in perceptions of science and technology between the Global North and South. This suggests that people’s perceptions of science and technology are primarily influenced by their perception of their own standard of living compared to that of their parents’ generation, regardless of their country’s economic status. Therefore, perceptions of socio-economic status in comparison to previous generations strongly shape attitudes towards scientific and technological advancements.

## Robustness verification

We have undertaken comprehensive robustness validations for our study. Initially, to ascertain that our findings are not solely the product of our selected estimation techniques, we executed a Multilevel Ordered Logit analysis. The outcomes of this are detailed in Supplementary Table C1 of Appendix C. These outcomes consistently align with our primary findings, suggesting the sturdiness of our analysis even when alternate methodologies are applied. Furthermore, our results maintain their qualitative integrity, irrespective of the omission or inclusion of control variables, as illustrated in Supplementary Tables C2–C5. Additionally, to guarantee that the findings from our comparative examination are not swayed by specific standout observations, we engaged in a jackknife analysis, excluding countries sequentially, as visualized in Supplementary Figures C1, C2. Lastly, a focused analysis on the direct association between income inequality and perceptions regarding the contributions of science and technology—bypassing the intermediary variable (perceptions of socioeconomic progression compared to the preceding generation)—reaffirms the negative relationship between the variables, as presented in Supplementary Figures C3, C4 and Supplementary Tables C6, C7. This consistency underlines the reliability of our results.

## Conclusion

In the contemporary era, marked by escalating societal inequalities, understanding the interplay between income disparities, perceived living standards, and their collective influence on perspectives regarding science and technology emerges as a research imperative. This study introduces the perceived living standard compared to previous generations as a potential intermediary variable, exploring its role in bridging the relationship between income inequality and public opinions regarding the relevance of scientific and technological advancements.

The methodological apparatus of our research encountered challenges rooted in data constraints, potentially compromising the strength of empirical substantiation. It is pivotal to underscore that our analytical framework predominantly hinged on variables extracted from the WVS Wave 7, inherently narrowing the investigative ambit. While we incorporated mechanisms to account for potential country-specific variations through the inclusion of country fixed effects, the contemporaneous nature of WVS Wave 7 with the COVID-19 pandemic infuses our analysis with a time-specific bias, which we could not rectify within this study’s confines. To surmount this hindrance, we endorse subsequent research endeavors to leverage expansive time-series datasets that encapsulate a more diverse set of nations, which could proffer richer insights into these interrelationships.

Despite inherent constraints, our study carves out a niche by elucidating the intricate relationships intertwining income inequality, perceptions about living standards, and the populace’s disposition towards science and technology. On a macroscopic scale, we spotlight the ramifications of income discrepancies on perceived living standards, especially in juxtaposition with antecedent generations. At a more granular, microscopic level, our study delves into how these ingrained perceptions sculpt societal views on the significance of science and technology. Both analytical prisms converge on the insight that escalating income disparities lead to a perceived deterioration in living standards when benchmarked against one’s parents, which consequentially dims the perceived prominence of science and technology in driving societal progress.

Given the context that escalating inequality can obstruct the formation of social consensus on various societal agendas (Kirklad et al., 2023; Han and Kwon, 2023b), our findings elucidate novel insights into the dynamics between inequality and societal divisions, specifically focusing on public discord concerning science and technology. Our exploration offers a fresh perspective on how such inequalities can drive divergent opinions and attitudes towards advancements in these fields, highlighting a nuanced interrelationship between disparity and societal discordance. This enhanced understanding could facilitate more informed discussions and interventions aimed at mitigating the divisive impacts of inequality on societal perspectives regarding science and technology.

Building on the foundational literature positing that economic disparity modulates political behavior through relational comparisons (Newman et al., 2015; Han and Kwon, 2023a), our research advances the discourse by postulating that such inequalities can foster adversarial perceptions about science and technology, especially when gauged against generational benchmarks. Our insights complement extant literature, which dissects divisive views on science and technology through various lenses such as socio-economic stratification (Baker and Merkely, 2023), media narratives (Gurevich, 2022), political factors and religious convictions (Akin et al., 2021), by introducing the salient variable of income inequality.

In this respect, this investigation offers a fresh perspective by outlining a conceptual scaffold, wherein income inequality shapes subjective outlooks *via* generational comparisons, potentially fostering skepticism regarding the contributions of science and technology. Our results affirm the hypothesis that mounting inequalities can distort an individual’s subjective socioeconomic evaluations, especially when referenced against preceding generations. Such warped perceptions, intensified by attenuated self-efficacy in an increasingly challenging societal context, may act as deterrents in fully embracing the potential of science and technology. As a corollary, the current study enriches the discourse on the widening schism in public sentiment towards scientific and technological advancements. Intriguingly, the delineated phenomena remain consistent across both the Global North and South, facilitating a nuanced understanding of the multifaceted public responses to pivotal events, such as the COVID-19 pandemic.

In conclusion, this study suggests that escalating economic inequality can exacerbate the public’s polarization regarding science and technology, potentially fueling further social rifts. Consequently, our results indicate that heightened inequality might deepen societal and political divisions on a spectrum of science and technology issues, encompassing areas such as pandemic response, the proliferation of artificial intelligence, and climate change considerations.

## Data availability statement

The original contributions presented in the study are included in the article/Supplementary material, further inquiries can be directed to the corresponding author.

## Ethics statement

Ethical review and approval was not required for the study on human participants in accordance with the local legislation and institutional requirements. Written informed consent from the participants was not required to participate in this study in accordance with the national legislation and the institutional requirements.

## Author contributions

SH and YL designed the research, analyzed and interpreted the data, and wrote the paper. All authors contributed to the article and approved the submitted version.
