# Supplementary material for: When I feel that I am better off, science seems to make the world better off too: inequality, perceived standard of living and perceptions toward science
Source: Front Psychol. 2023 Oct 5;14:1202550. doi: 10.3389/fpsyg.2023.1202550 (PMC10585155; doi:10.3389/fpsyg.2023.1202550)
Supplement: Supplementary file 1 [file Table_1.pdf]

When I Feel That I am Better off, Science Seems to Make the World Better Off Too

Online appendix

Tables

Table A1. Data source 3

Table A2. Variables (WVS) 3

Table A3. Descriptive statistics 4

Table B1. Perceived standard of living and Science/Technology 7

Table B2. Perceived standard of living and Science/Technology by the Global North and the Global South 1 8

Table B3. Perceived standard of living and Science/Technology by the Global North and the Global South 2 9

Table C1. Perceived standard of living and Science/Technology (Multilevel ordered logit)........10

Table C2. Perceived standard of living and Science/Technology (alternative models 1).............11

Table C3. Perceived standard of living and Science/Technology (alternative models 2).............12

Table C4. Perceived standard of living and Science/Technology (alternative models 3).............13

Table C5. Perceived standard of living and Science/Technology (alternative models 4).............14

Table C6. Income inequality and Better off because of science and technology.............................17

Table C7. Income inequality and More opportunities because of Science and Technology 19

Figures

Figure B1. Kernel Density Estimation of the perceived standard of living by group based on income inequality 5
Figure B2. t-test results by Better and Worse 6

Figure C1. Jackknife analysis 1 (Perceived standard of living and Better off because of Science and Technology) 15
Figure C2. Jackknife analysis 2 (Perceived standard of living and More opportunities because of Science and Technology) 15
Figure C3. Income inequality and Better off because of science and technology 16
Figure C4. Income inequality and More opportunities because of Science and Technology......... 18

Appendix A

Table A1. Data source

Data	Source	Website
Income Inequality	World Inequality Database	https://wid.world/data/
GDP per capita	Maddison Project Database	https://www.rug.nl/ggdc/historical development/maddison/?lang=en
World Values Surveys (WVS)	World Values Survey Association	https://www.worldvaluessurvey.org/wvs.jsp

Table A2. Variables (WVS)

	Variable	Description	Note
1	Better off	Perceived standard of living<br><br>Q: Comparing your standard of living with your parents' standard of living when they were about your age, would you say that you are better off or worse off?<br><br>A: Worse off (=1), About the same (=2), Better off (=3)	
2	Better off because of Science and Technology	Q: The world is better off or worse off because of science and technology.<br><br>A: A lot worse off (=1) ~ A lot better off (=10)	
3	More opportunities because of Science and Technology	Q: There will be more opportunities for the next generation because of science and technology.<br><br>A: Completely disagree (=1) ~ Completely agree (=10)	
4	Income	Income scale: 1 ~ 10	
5	Education	Lower (=1) ~ Upper (=3)	
6	Sex	Female = 1<br>Otherwise = 0	
7	Age	Age scale: 1 ~ 6	
8	Marital	Living with a spouse (or a partner) = 1	

		Otherwise = 0
9	Urban	Size scale: 1 ~ 8
10	Religious	Religious = 1<br>Otherwise = 0
11	Political orientation	Left (=1) ~ Right (=10)

Table A3. Descriptive statistics

	N	Mean	Std dev	Min	Median	Max
Gini index	46	0.572	0.073	0.429	0.565	0.748
Top 1% share	46	0.164	0.039	0.088	0.166	0.281
Top 10% share	46	0.457	0.076	0.326	0.454	0.649
Top 10/Bottom 50	46	3.627	1.943	1.464	3.083	10.44
GDP per capita	46	18300	14153	1611	13323	55334
Perceived standard of living	42845	2.385	0.749	1	3	3
Better off because of Science and Technology	42845	6.949	2.526	1	7	10
More opportunities because of Science and Technology	42845	7.428	2.365	1	7	10
Income	42845	4.857	2.09	1	5	10
Education	42845	2.009	0.808	1	2	3
Sex	42845	0.512	0.499	0	1	1
Age	42845	2.879	1.575	1	3	6
Marital	42845	0.633	0.481	0	1	1
Urban	42845	5.188	2.461	1	6	8
Religious	42845	0.638	0.48	0	1	1
Political orientation	42845	5.731	2.487	1	5	10

Appendix B

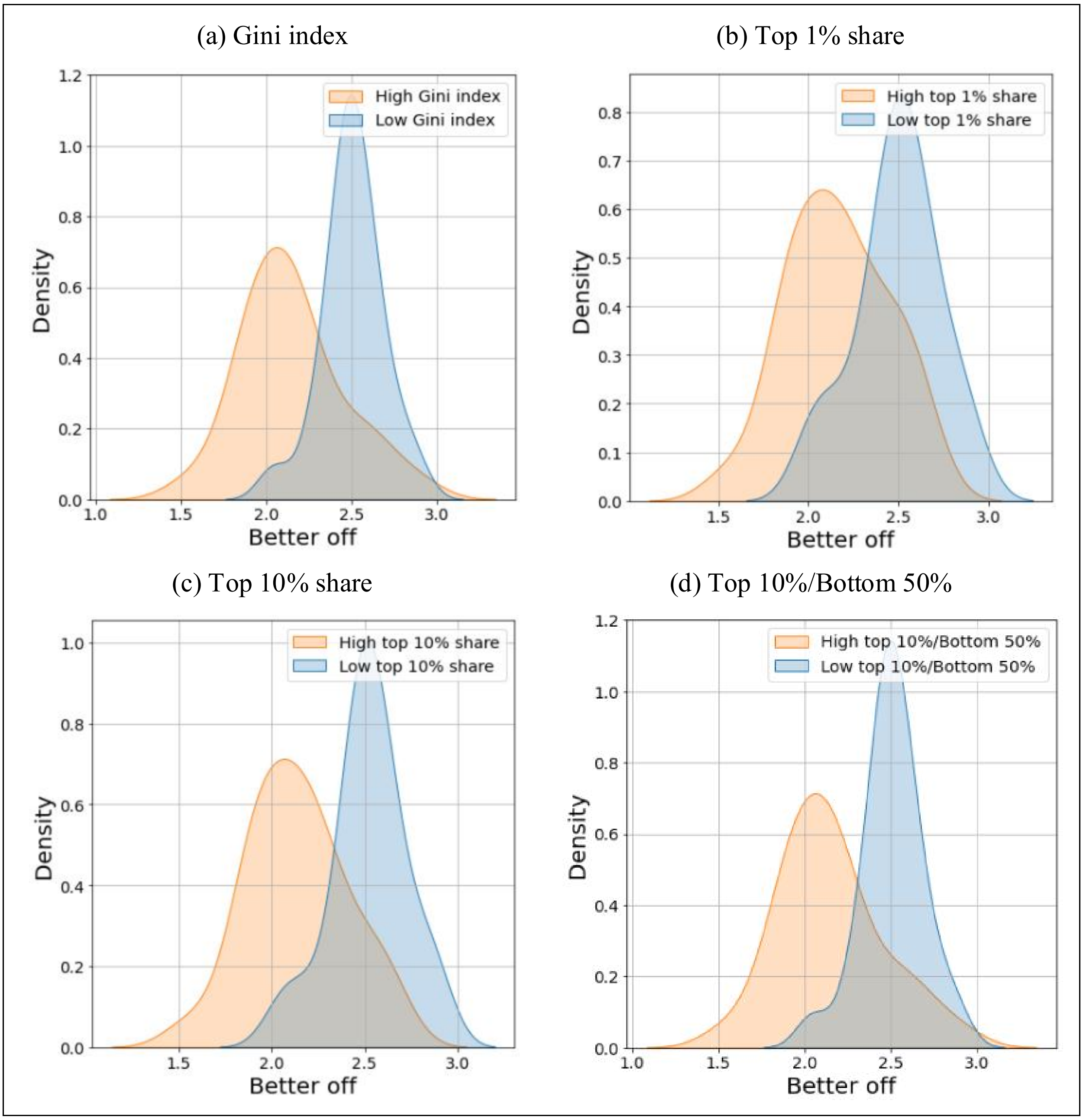

Figure B1. Kernel Density Estimation of the perceived standard of living by group based on income inequality

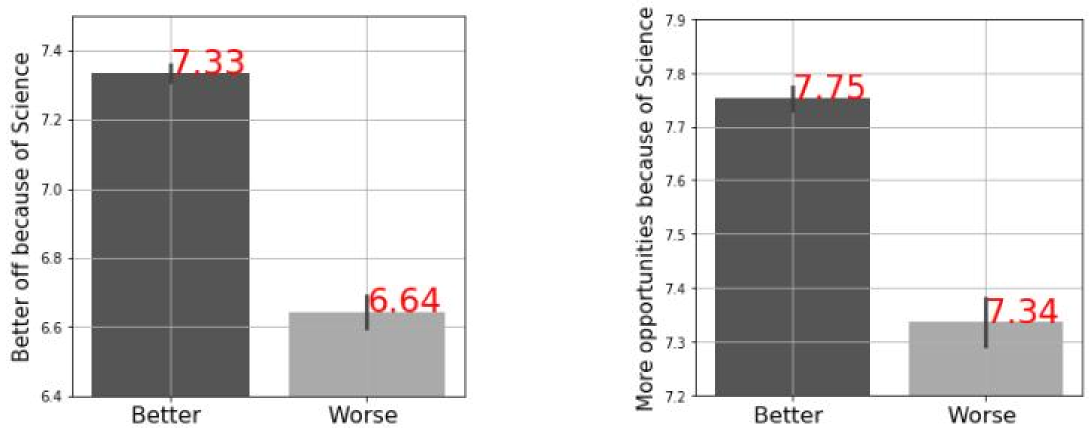

$t = 26.1288$	$P = 0.0000$	$t = 17.0601$	$P = 0.0000$
Note: See Table A9 in Appendix A for full results; Source: 2004 and 2014 ISSP			

Figure B2. *t*-test results by Better and Worse

Table B1. Perceived standard of living and Science/Technology

	DV: Better off because of Science and Technology		DV: More opportunities because of Science and Technology	
	(1)	(2)	(3)	(4)
Worse	-0.259***		-0.246**	
	(0.051)		(0.065)	
Better	0.208***		0.19***	
	(0.049)		(0.031)	
Better off		0.229***		0.213***
		(0.026)		(0.027)
Income	0.06***	0.06***	0.038**	.038**
	(0.011)	(0.011)	(0.012)	(0.012)
Education	0.131**	0.131**	0.063	0.063
	(0.041)	(0.042)	(0.038)	(0.038)
Age	0.011	0.011	0.0002	0.0001
	(0.017)	(0.017)	(0.012)	(0.012)
Sex	-0.174***	-0.174***	-0.111**	-0.111**
	(0.044)	(0.044)	(0.034)	(0.034)
Marital	-0.053	-0.053	-0.025	-0.025
	(0.046)	(0.046)	(0.035)	(0.035)
Urban	0.011	0.011	0.011	0.011
	(0.009)	(0.009)	(0.012)	(0.012)
Religious	-0.137*	-0.137*	-0.065	-0.065
	(0.06)	(0.06)	(0.043)	(0.043)
Political orientation	0.053**	0.053**	0.06***	0.061***
	(0.015)	(0.015)	(0.013)	(0.013)
Country FE	√	√	√	√
Intercept	5.963***	5.488***	6.53***	6.086***
	(0.129)	(0.131)	(0.16)	(0.135)
N	42845	42845	42808	42808

Country clustered standard errors in parentheses: *p < 0.05, ** p < 0.01, *** p < 0.001

Table B2. Perceived standard of living and Science/Technology by the Global North and the Global South 1

	DV: Better off because of Science and Technology		DV: More opportunities because of Science and Technology	
	Global North	Global South	Global North	Global South
Worse	-0.217**	-0.269***	-0.273**	-0.241**
	(0.061)	(0.065)	(0.102)	(0.08)
Better	0.281**	0.177**	0.253***	0.165***
	(0.063)	(0.061)	(0.048)	(0.037)
Income	0.075**	0.055**	0.016	0.045*
	(0.015)	(0.013)	(0.013)	(0.016)
Education	0.165	0.119**	0.006	0.082*
	(0.119)	(0.037)	(0.096)	(0.037)
Age	0.068	-0.008	0.002	0.001
	(0.04)	(0.018)	(0.027)	(0.012)
Sex	-0.171*	-0.177**	-0.087	-0.128**
	(0.081)	(0.052)	(0.055)	(0.042)
Marital	0.089	-0.117*	0.058	-0.049
	(0.051)	(0.056)	(0.051)	(0.044)
Urban	0.025*	0.008	0.041**	0.003
	(0.011)	(0.012)	(0.011)	(0.015)
Religious	-0.15	-0.113	-0.013	-0.071
	(0.077)	(0.078)	(0.046)	(0.058)
Political orientation	-0.052	0.077***	-0.036	0.083***
	(0.032)	(0.012)	(0.023)	(0.012)
Country FE	√	√	√	√
Intercept	6.949***	6.024***	6.831***	6.401***
	(0.272)	(0.153)	(0.29)	(0.198)
N	12592	30253	12596	30212

Country clustered standard errors in parentheses: *p < 0.05, ** p < 0.01, *** p < 0.001

Table B3. Perceived standard of living and Science/Technology by the Global North and the Global South 2

	DV: Better off because of Science and Technology		DV: More opportunities because of Science and Technology	
	Global North	Global South	Global North	Global South
Better off	0.256***	0.215***	0.261***	0.196***
	(0.049)	(0.03)	(0.051)	(0.032)
Income	0.075**	0.055**	0.016	0.045**
	(0.015)	(0.014)	(0.013)	(0.016)
Education	0.166	0.118**	0.006	0.081**
	(0.12)	(0.038)	(0.096)	(0.037)
Age	0.068	-0.008	0.002	0.0008
	(0.04)	(0.018)	(0.027)	(0.012)
Sex	-0.171*	-0.177**	-0.087	-0.128**
	(0.081)	(0.052)	(0.055)	(0.042)
Marital	0.089	-0.117*	0.058	-0.049
	(0.052)	(0.056)	(0.051)	(0.044)
Urban	0.025*	0.008	0.04**	0.003
	(0.011)	(0.012)	(0.011)	(0.015)
Religious	-0.15*	-0.112	-0.013	-0.071
	(0.077)	(0.078)	(0.046)	(0.058)
Political orientation	-0.052	0.077***	-0.036	0.083***
	(0.032)	(0.012)	(0.023)	(0.012)
Country FE	√	√	√	√
Intercept	6.454***	5.565***	6.303***	5.984***
	(0.285)	(0.154)	(0.328)	(0.159)
N	12592	30253	12596	30212

Country clustered standard errors in parentheses: *p < 0.05, ** p < 0.01, *** p < 0.001

Appendix C

Table C1. Perceived standard of living and Science/Technology (Multilevel ordered logit)

	DV: Better off because of Science and Technology		DV: More opportunities because of Science and Technology	
	(1)	(2)	(3)	(4)
Worse	-0.181***<br>(0.027)		-0.181***<br>(0.027)	
Better	0.187***<br>(0.02)		0.157***<br>(0.02)	
Better off		0.184***<br>(0.012)		0.167***<br>(0.012)
Income	0.038***<br>(0.004)	0.038***<br>(0.004)	0.019***<br>(0.004)	0.019***<br>(0.004)
Education	0.084***<br>(0.012)	0.085***<br>(0.012)	0.035**<br>(0.012)	0.035**<br>(0.012)
Age	0.014*<br>(0.006)	0.014*<br>(0.006)	0.005<br>(0.006)	0.005<br>(0.006)
Sex	-0.142***<br>(0.017)	-0.142***<br>(0.017)	-0.109***<br>(0.017)	-0.109***<br>(0.017)
Marital	-0.035<br>(0.018)	-0.035<br>(0.018)	-0.03<br>(0.018)	-0.03<br>(0.018)
Urban	0.008<br>(0.004)	0.008<br>(0.004)	0.008<br>(0.004)	0.008<br>(0.004)
Religious	-0.116***<br>(0.02)	-0.116***<br>(0.02)	-0.048**<br>(0.021)	-0.048**<br>(0.021)
Political orientation	0.051***<br>(0.003)	0.051***<br>(0.003)	0.06***<br>(0.003)	0.06***<br>(0.003)
Random intercept	0.298<br>(0.069)	0.298<br>(0.069)	0.238<br>(0.055)	0.237<br>(0.055)
Wald Chi^2	755.43***	755.42***	564.03***	563.68***
N	42845	42845	42808	42808

Standard errors in parentheses: *p < 0.05, ** p < 0.01, *** p < 0.001

Table C2. Perceived standard of living and Science/Technology (alternative models 1)

	DV: Better off because of Science and Technology					
	(1)	(2)	(3)	(4)	(5)	(6)
Worse	-0.276***	-0.245***	-0.248***	-0.244***	-0.251***	-0.25***
	(0.056)	(0.055)	(0.056)	(0.056)	(0.056)	(0.054)
Better	0.232***	0.207***	0.199***	0.196***	0.191***	0.191***
	(0.048)	(0.048)	(0.048)	(0.047)	(0.047)	(0.048)
Income		0.07***	0.06***	0.06***	0.058***	0.058***
		(0.012)	(0.01)	(0.01)	(0.01)	(0.01)
Education			0.124**	0.128**	0.112**	0.111**
			(0.034)	(0.036)	(0.031)	(0.032)
Age				0.008	0.006	0.01
				(0.017)	(0.015)	(0.016)
Sex					-0.136***	-0.131***
					(0.035)	(0.035)
Marital					-0.042	-0.038
					(0.036)	(0.037)
Urban					0.005	0.003
					(0.008)	(0.008)
Religious						-0.104**
						(0.049)
Political orientation						
Country FE	√	√	√	√	√	√
Intercept	6.81***	6.384***	6.181***	6.145***	6.268***	6.309***
	(0.03)	(0.067)	(0.111)	(0.136)	(0.117)	(0.116)
N	68790	67413	66968	66717	65663	64320

Country clustered standard errors in parentheses: *p < 0.05, ** p < 0.01, *** p < 0.001

Table C3. Perceived standard of living and Science/Technology (alternative models 2)

	DV: Better off because of Science and Technology					
	(1)	(2)	(3)	(4)	(5)	(6)
Better off	0.25***<br>(0.026)	0.223***<br>(0.026)	0.219***<br>(0.027)	0.216***<br>(0.026)	0.216***<br>(0.027)	0.215***<br>(0.027)
Income		0.07***<br>(0.012)	0.06***<br>(0.011)	0.06***<br>(0.01)	0.058***<br>(0.01)	0.058***<br>(0.01)
Education			0.123**<br>(0.034)	0.127**<br>(0.036)	0.112**<br>(0.032)	0.11**<br>(0.032)
Age				0.008<br>(0.017)	0.005<br>(0.015)	0.01<br>(0.016)
Sex					-0.136***<br>(0.035)	-0.131***<br>(0.035)
Marital					-0.042<br>(0.036)	-0.038<br>(0.037)
Urban					0.005<br>(0.008)	0.003<br>(0.008)
Religious						-0.104**<br>(0.049)
Political orientation						
Country FE	√	√	√	√	√	√
Intercept	6.294***<br>(0.056)	5.924***<br>(0.079)	5.726***<br>(0.119)	5.698***<br>(0.146)	5.816***<br>(0.129)	5.86***<br>(0.127)
N	68790	67413	66968	66717	65663	64320

Country clustered standard errors in parentheses: *p < 0.05, ** p < 0.01, *** p < 0.001

Table C4. Perceived standard of living and Science/Technology (alternative models 3)

	DV: More opportunities because of Science and Technology					
	(1)	(2)	(3)	(4)	(5)	(6)
Worse	-0.202**	-0.187**	-0.188**	-0.186**	-0.189**	-0.191**
	(0.058)	(0.059)	(0.06)	(0.06)	(0.061)	(0.059)
Better	0.193***	0.176***	0.172***	0.167***	0.158***	0.151***
	(0.031)	(0.031)	(0.032)	(0.031)	(0.029)	(0.03)
Income		0.036**	0.031**	0.031**	0.031**	0.032**
		(0.011)	(0.011)	(0.011)	(0.011)	(0.011)
Education			0.058*	0.063*	0.059*	0.059*
			(0.029)	(0.029)	(0.029)	(0.029)
Age				0.01	0.012	0.012
				(0.011)	(0.011)	(0.012)
Sex					-0.072**	-0.069**
					(0.028)	(0.029)
Marital					-0.03	-0.031
					(0.031)	(0.031)
Urban					-0.0007	-0.001
					(0.009)	(0.009)
Religious						0.017
						(0.043)
Political orientation						
Country FE	√	√	√	√	√	√
Intercept	7.245***	6.97***	6.869***	6.825***	6.894***	6.896***
	(0.02)	(0.064)	(0.089)	(0.096)	(0.112)	(0.107)
N	68663	67293	66854	66604	65546	64195

Country clustered standard errors in parentheses: *p < 0.05, ** p < 0.01, *** p < 0.001

Table C5. Perceived standard of living and Science/Technology (alternative models 4)

	DV: More opportunities because of Science and Technology					
	(1)	(2)	(3)	(4)	(5)	(6)
Better off	0.197***<br>(0.029)	0.181***<br>(0.029)	0.178***<br>(0.029)	0.175***<br>(0.029)	0.171***<br>(0.028)	0.167***<br>(0.028)
Income		0.036**<br>(0.011)	0.031**<br>(0.011)	0.031**<br>(0.011)	0.031**<br>(0.011)	0.032**<br>(0.011)
Education			0.058<br>(0.029)	0.063*<br>(0.029)	0.058*<br>(0.029)	0.059*<br>(0.029)
Age				0.01<br>(0.011)	0.012<br>(0.011)	0.011<br>(0.012)
Sex					-0.072*<br>(0.028)	-0.069*<br>(0.029)
Marital					-0.03<br>(0.031)	-0.031<br>(0.031)
Urban					-0.0007<br>(0.009)	-0.001<br>(0.009)
Religious						0.017<br>(0.043)
Political orientation						
Country FE	√	√	√	√	√	√
Intercept	6.848***<br>(0.062)	6.604***<br>(0.082)	6.506***<br>(0.096)	6.468***<br>(0.101)	6.542***<br>(0.11)	6.548***<br>(0.103)
N	68663	67293	66854	66604	65546	64195

Country clustered standard errors in parentheses: *p < 0.05, ** p < 0.01, *** p < 0.001

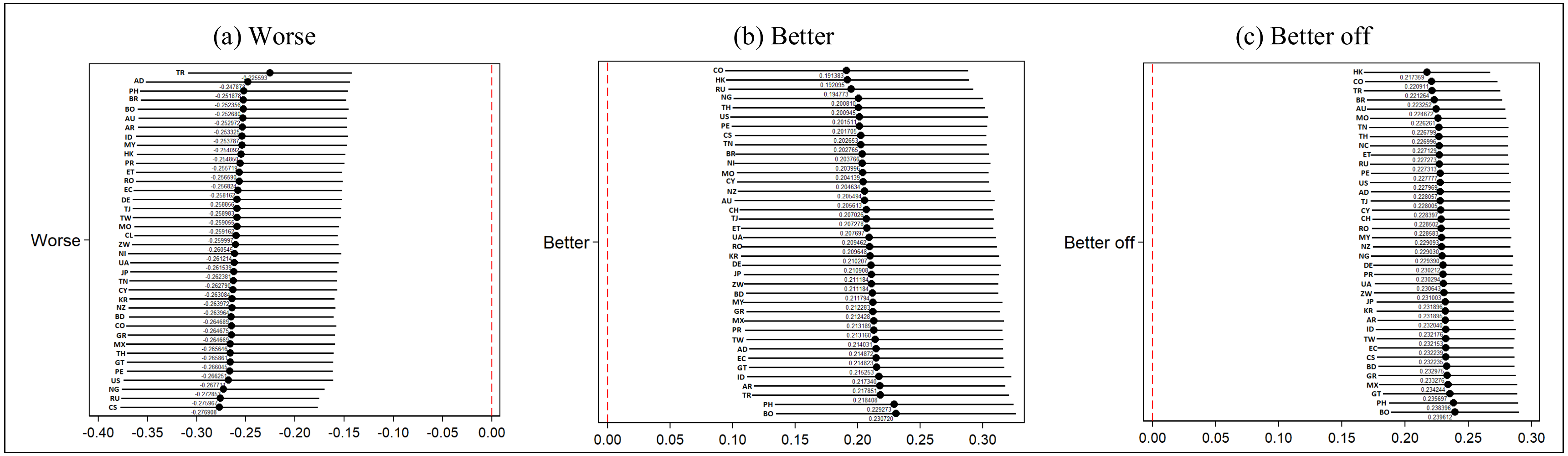

Figure C1. Jackknife analysis 1 (Perceived standard of living and Better off because of Science and Technology)

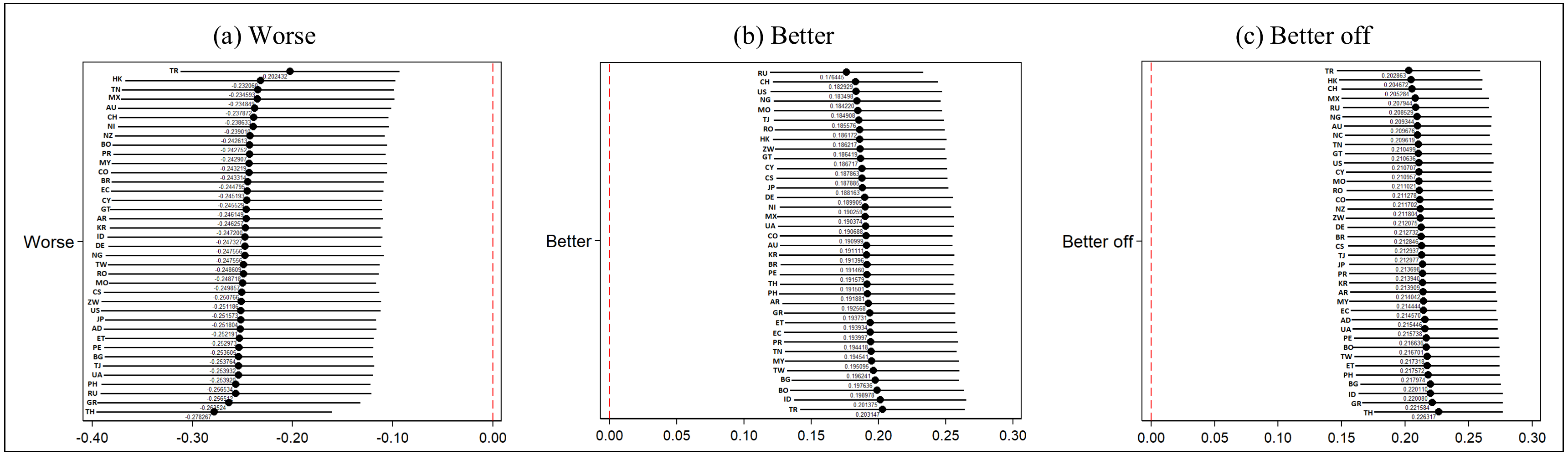

Figure C2. Jackknife analysis 2 (Perceived standard of living and More opportunities because of Science and Technology)

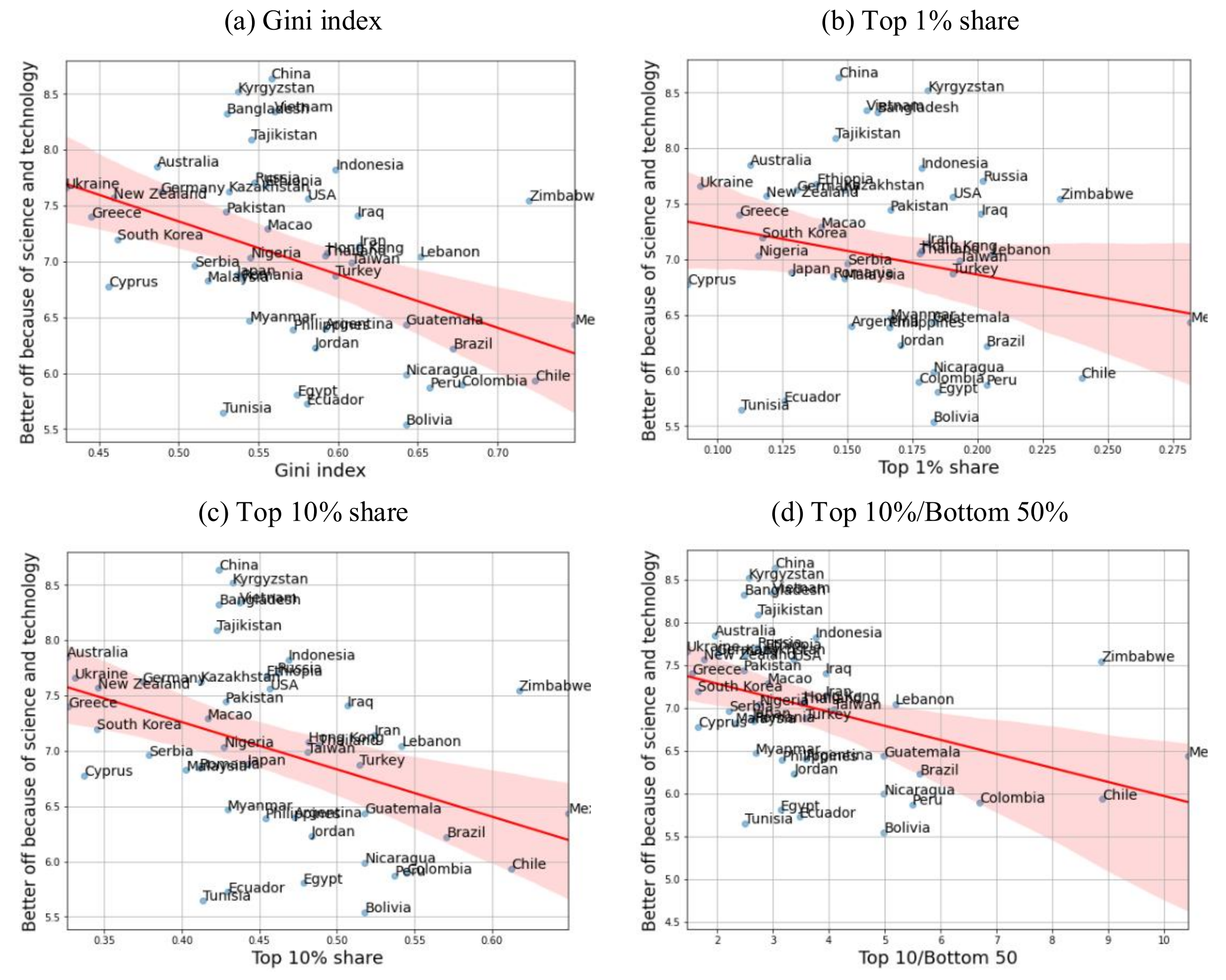

Source: Better off because of science and technology from WVS Wave 7; Income inequality variables are from World Inequality Database

Figure C3. Income inequality and Better off because of science and technology

Table C6. Income inequality and Better off because of science and technology

	DV: Better off because of Science and Technology			
	(1)	(2)	(3)	(4)
Gini index	-5.171***<br>(1.134)			
Top 1% share		-4.434*<br>(2.566)		
Top 10% share			-4.703***<br>(1.112)	
Top10/Bottom50				-2.555**<br>(0.794)
ln GDP p.c.	-0.152<br>(0.123)	-0.069<br>(0.13)	-0.153<br>(0.125)	-0.396<br>(0.063)
Intercept	11.418***<br>(1.458)	8.398***<br>(1.423)	10.627***<br>(1.438)	12.804***<br>(0.792)
R^2	0.21	0.04	0.17	0.33
N	46	46	46	46

Note: Country clustered standard errors in parentheses: *p < 0.05, ** p < 0.01, *** p < 0.001

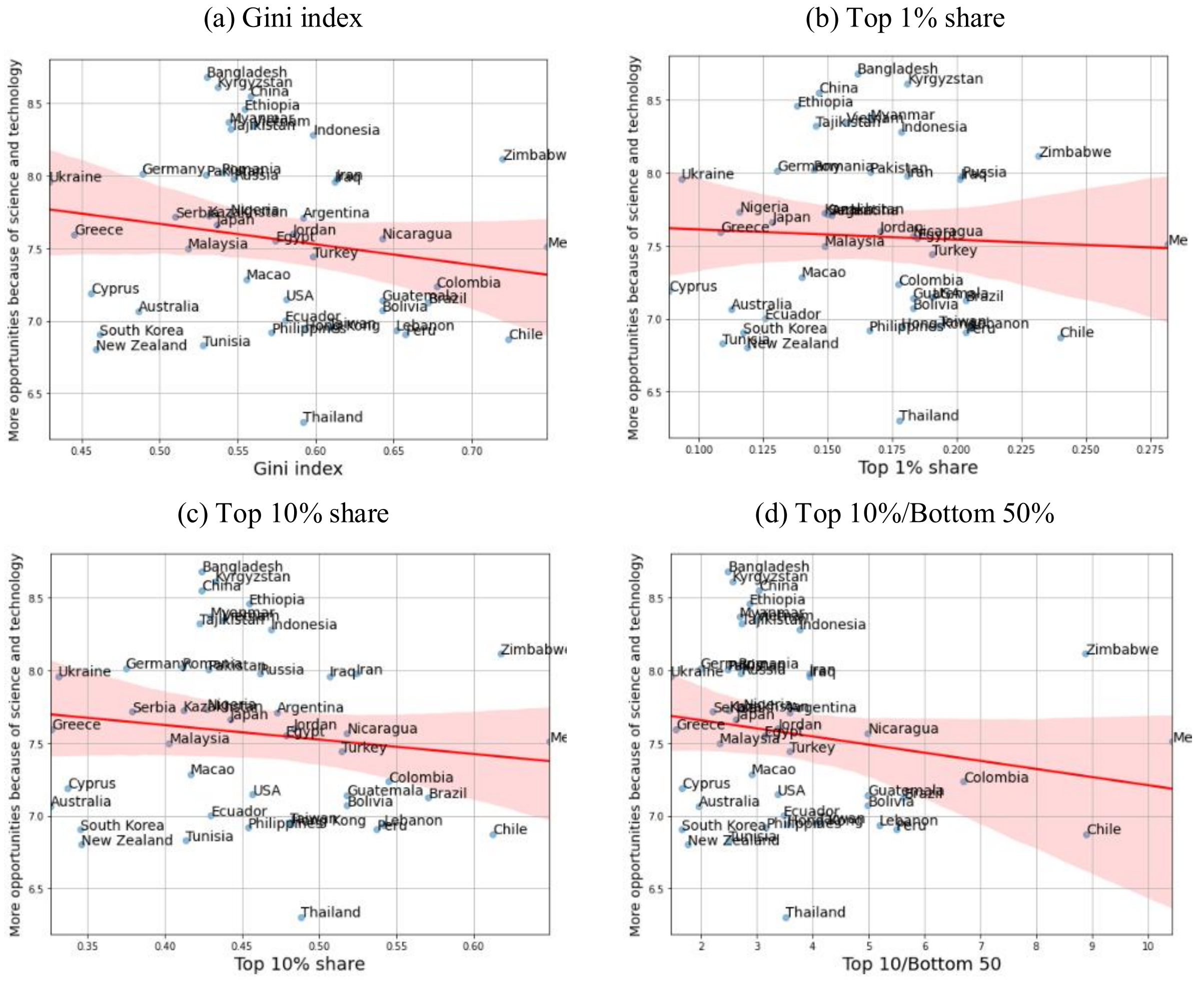

Source: More opportunities because of science and technology from WVS Wave 7; Income inequality variables are from World Inequality Database

Figure C4. Income inequality and More opportunities because of Science and Technology

Table C7. Income inequality and More opportunities because of Science and Technology

	DV: More opportunities because of Science and Technology			
	(1)	(2)	(3)	(4)
Gini index	-2.555**<br>(0.794)			
Top 1% share		-1.905<br>(1.592)		
Top 10% share			-2.186**<br>(0.776)	
Top10/Bottom50				-0.091*<br>(0.034)
ln GDP p.c.	-0.396<br>(0.063)	-0.353<br>(0.071)	-0.393<br>(0.068)	-0.384<br>(0.067)
Intercept	12.804***<br>(0.792)	11.245***<br>(0.762)	12.318***<br>(0.804)	11.567***<br>(0.685)
R^2	0.33	0.25	0.31	0.32
N	46	46	46	46

Note: Country clustered standard errors in parentheses: *p < 0.05, ** p < 0.01, *** p < 0.001
